# Morphometric Parameters of the Distal End of the Femur: A Magnetic Resonance Imaging Study

**DOI:** 10.7759/cureus.85481

**Published:** 2025-06-06

**Authors:** Ritu Roy, Navbir Pasricha, Swagat Mahapatra, Rajan Bhatnagar, Eti Sthapak, Shamrendra Narayan, Anamika Gaharwar

**Affiliations:** 1 Department of Anatomy, Dr. Ram Manohar Lohia Institute of Medical Sciences, Lucknow, IND; 2 Department of Orthopedic Surgery, Dr. Ram Manohar Lohia Institute of Medical Sciences, Lucknow, IND; 3 Department of Radiodiagnosis, Dr. Ram Manohar Lohia Institute of Medical Sciences, Lucknow, IND

**Keywords:** femoral surface, knee osteoarthritis/koa, knee prosthesis survival, tibial surface, total knee arthroplasty (tka)

## Abstract

Background: Knee osteoarthritis (OA) is a chronic, progressive condition that significantly impairs quality of life. The femoral component of the total knee arthroplasty (TKA) implant is a key intervention for alleviating OA-related morbidity, requiring precise anatomical considerations to ensure effective outcomes.

Methods: This study aimed to document the morphometric parameters of the distal femur in the North Indian population using magnetic resonance imaging (MRI). It specifically focused on measuring these parameters in healthy adults and analyzing the variations based on age and sex. An observational study was conducted utilizing MRI scans of 70 healthy adults of both sexes.

Results: Morphometric parameters of femoral anteroposterior diameter, femoral posterior condylar length, femoral lateral anteroposterior distance, and femoral medial anteroposterior distance of distal end femur showed higher values in men compared with women (p < 0.001). Younger male patients displayed higher values than female patients, compared to male patients in the more than 30 years age group.

Conclusion: Significant gender differences were observed in the study, which highlights the importance of designing femoral components of TKA prostheses that account for anatomical variations of the particular population in question, to potentially improve implant stability and longevity for the patient.

## Introduction

The knee is the largest synovial joint in the human body, which consists of three separate compartments, making it a complex hinge joint. This arrangement helps in the transfer of body weight from the femur above to the talus below and offers a fulcrum for the powerful extensor and flexor muscles that act on the joint during propulsion [1]. The femur and tibia are not directly aligned, with the femur slanting inward, resulting in a normal valgus deviation of about 10°-12°. The distal femoral surface, with medial and lateral condyles separated by an intercondylar fossa, articulates with the proximal tibial surface, which features distinct medial and lateral condyles [1]. Osteoarthritis (OA) of the knee, a chronic, progressive condition, often affects the patellofemoral and tibiofemoral compartments, leading to decreased quality of life. Total knee arthroplasty (TKA) can alleviate OA-related morbidity and accurate prosthesis, and requires precise bone-cutting, soft-tissue balance, and implant coverage. It is crucial to develop a prosthesis design that accommodates anatomical variations across sexes and ethnicities. Accurate anthropometric data enhance implant stability and longevity [2-6].

Previous cadaveric studies show Indian women have smaller femurs and tibias compared to men and those of Western populations, values of which are used routinely to design implants for the Indian population as well. It needs to be emphasized that knee anatomy is further complicated in Indian women with ethnic sociocultural practices followed during household work. Mixed results in studies on gender-specific implants highlight the need to consider racial and morphotype differences in distal femur anatomy for better TKA outcomes [7-11].

This study aims to provide normative data on morphometric parameters of the distal end of the femur for the North Indian population, using magnetic resonance imaging (MRI), which can potentially be useful for designing and guiding the design of sex-specific and morphometrically suitable femoral components for TKA prostheses. The primary objective was to record these morphometric parameters while also investigating variations based on age and sex.

## Materials and methods

**Figure 1:**
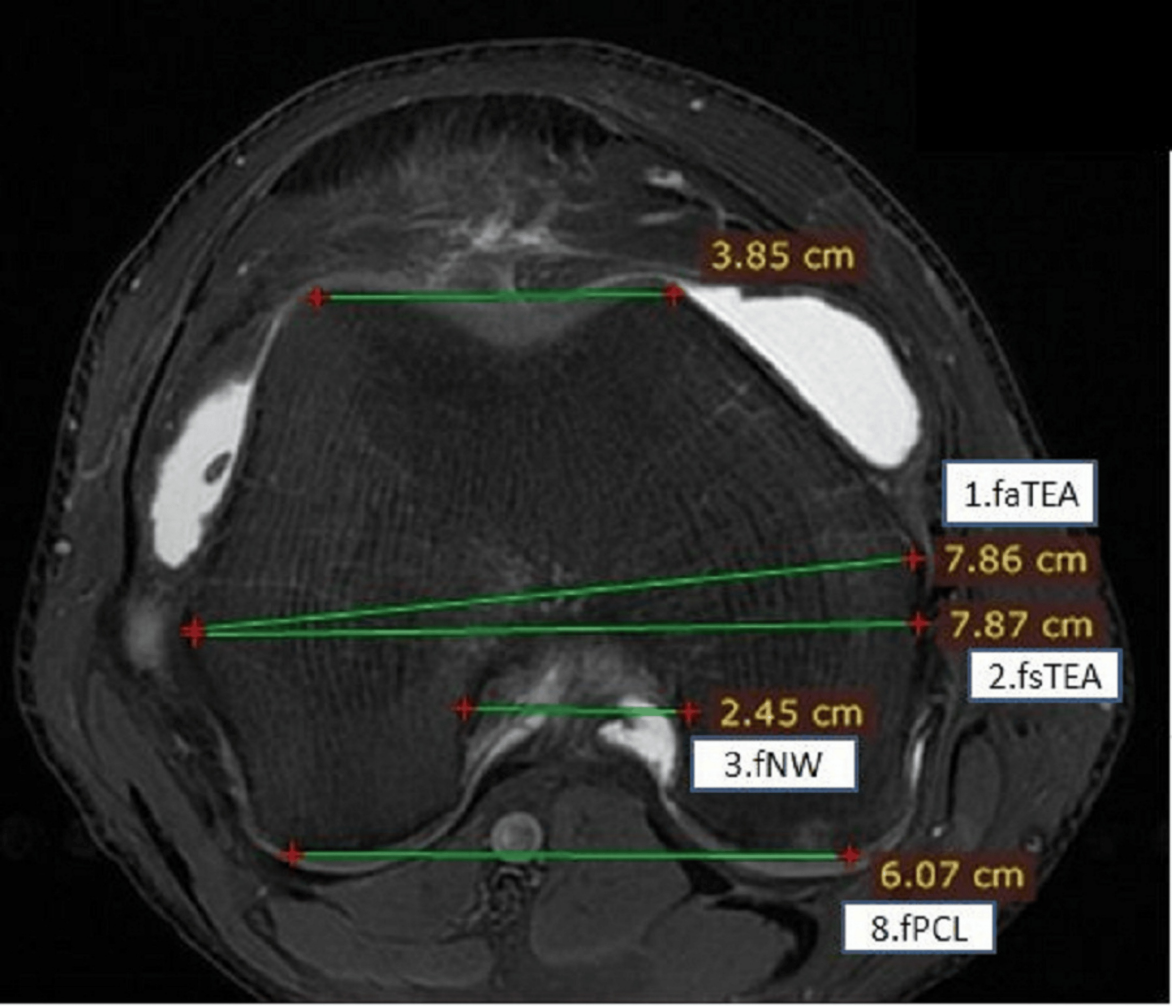
Measurements on the distal end of the femur in axial view showing femoral surgical and anatomical transepicondylar axis, femoral intercondylar notch width, and femoral posterior condylar length fsTEA: femoral surgical transepicondylar axis; faTEA: femoral anatomical transepicondylar axis; fNW: femoral intercondylar notch width; fPCL: femoral posterior condylar length

**Figure 2:**
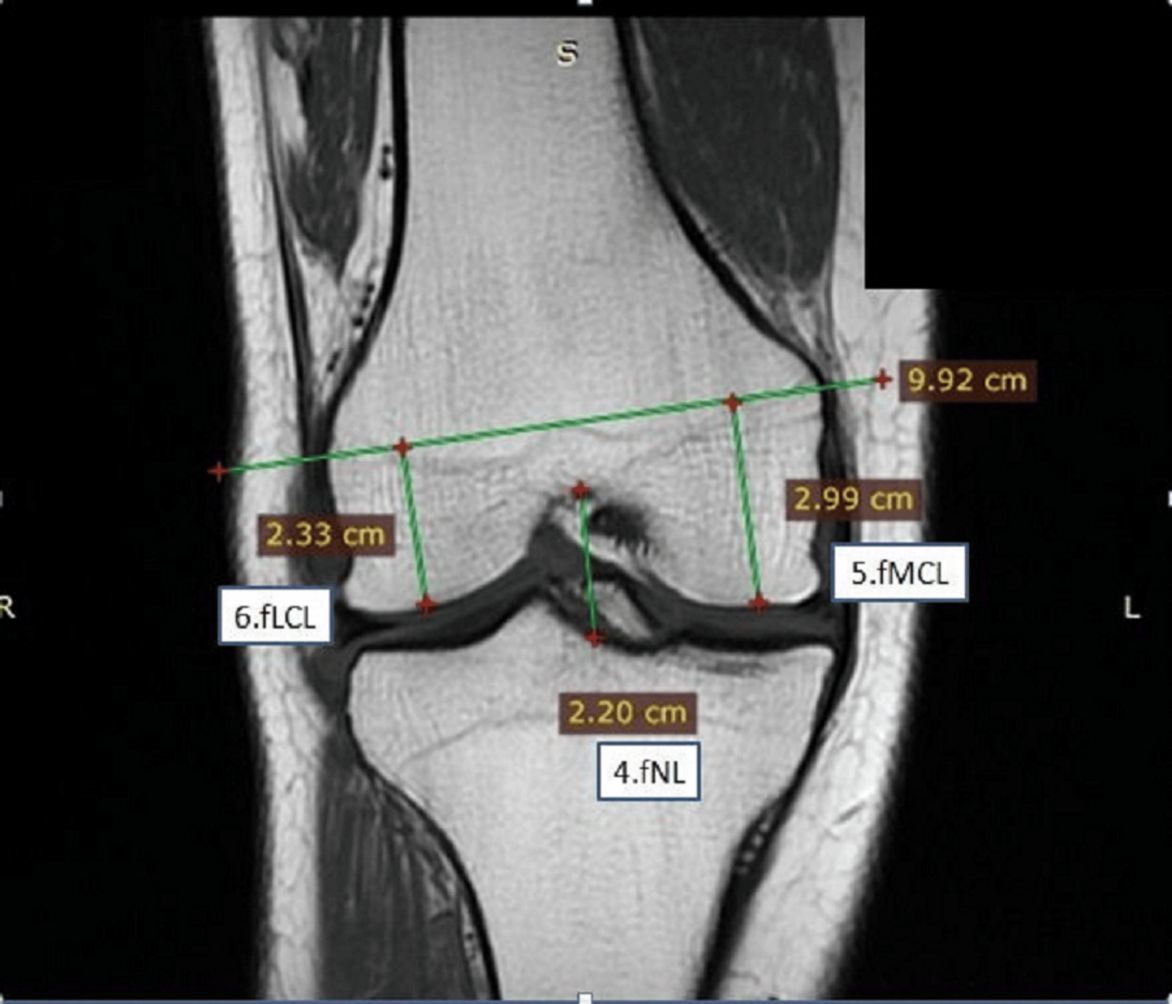
Measurements on the distal end of the femur in coronal view fNL: femoral notch length; fMCL: femoral medial condylar length; fLCL: femoral lateral condylar length

**Figure 3:**
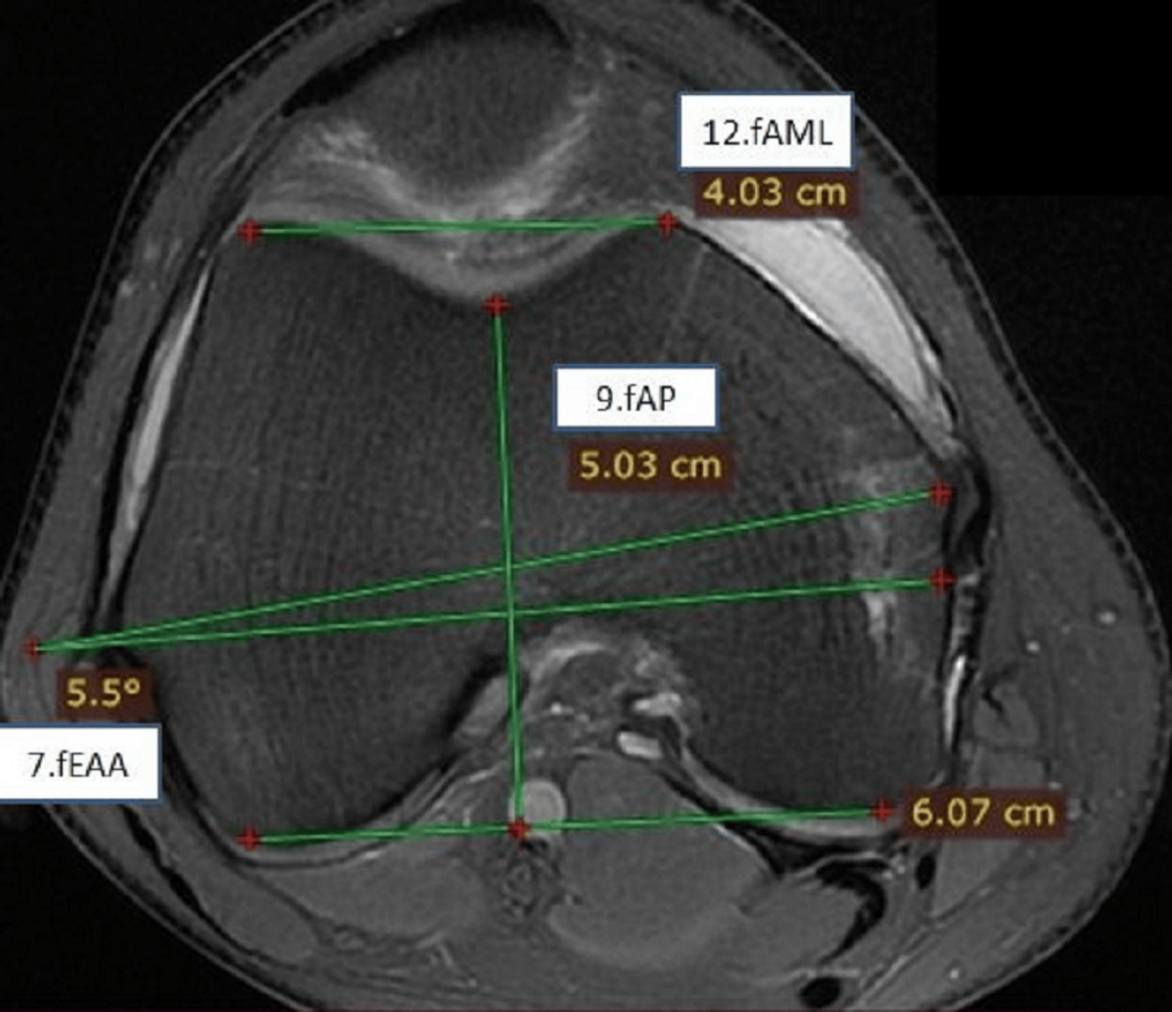
Measurements on the distal end of the femur in axial view showing femoral epicondylar axis angle, femoral anteroposterior length, and femoral antero-medio-lateral length fEAA: femoral epicondylar axis angle; fAP: femoral anteroposterior; fAML: femoral antero-medio-lateral

**Figure 4:**
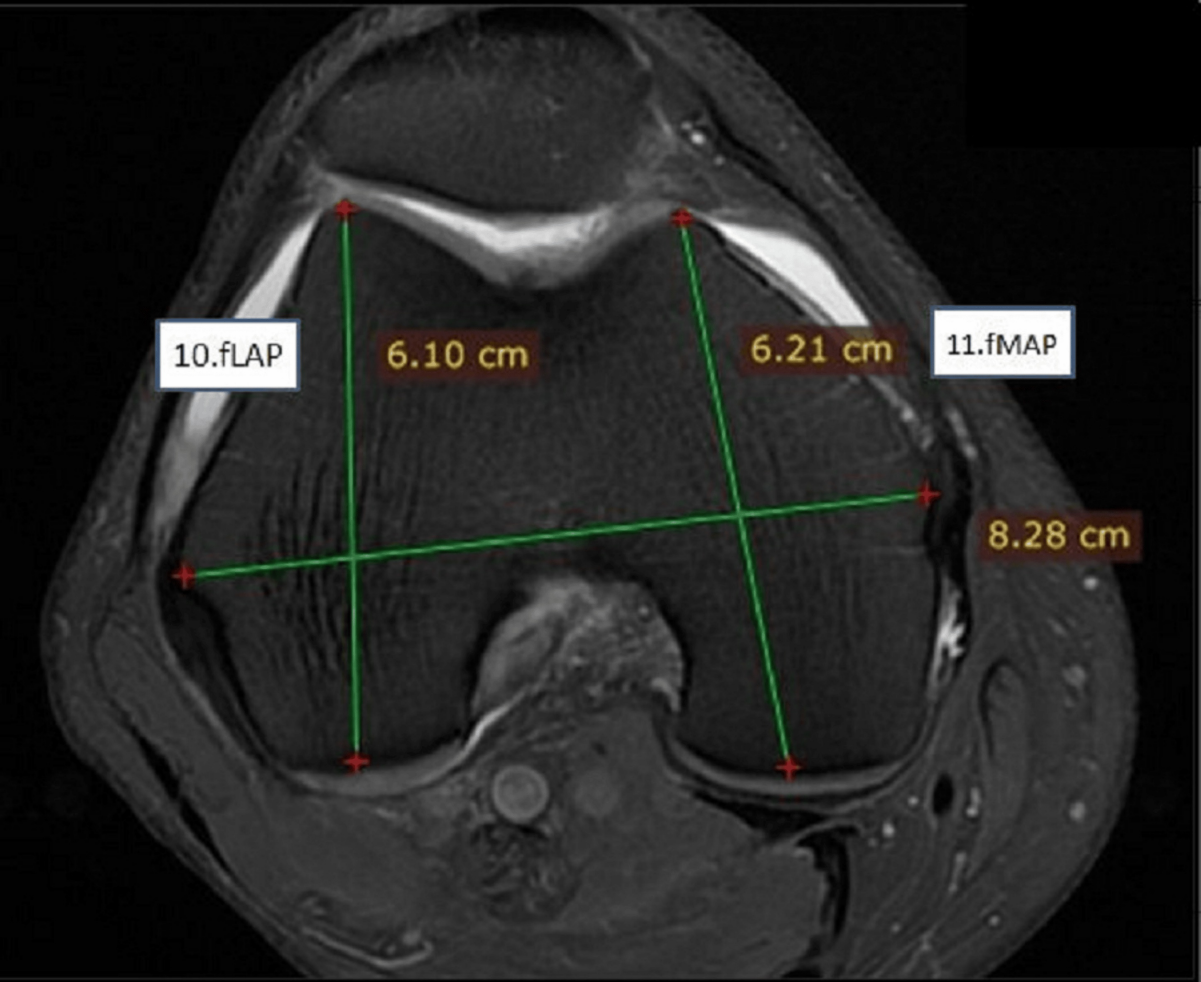
Measurements on the distal end of the femur in axial view showing femoral lateral anteroposterior and femoral medial anteroposterior lengths fLAP: femoral lateral anteroposterior; fMAP: femoral medial anteroposterior

A cross-sectional, observational study was conducted over an 18-month period in the Departments of Anatomy and Radiodiagnosis at a multispecialty referral institute primarily serving a north Indian population. The research aimed to perform morphometric measurements of the distal end of the femur (parameters are listed in Table 1 and illustrated in Figures 1-4) using normal MRI scans of the knee from 35 male and 35 female subjects, all aged 18 years and older. Participants were selected based on the inclusion criteria of being part of the Indian population with normal bones as evidenced by their MRI scans. Individuals with marked osteoarthritic changes in the distal end of the femur or a history of fractures or other bony pathologies in that region were excluded. Informed consent was obtained from patients before noting the demographic details and MRI scanning.

**Table 1:** Measurements taken for distal end of femur (f) Measurements were taken as described in [12-15]

Parameters	Abbreviation	Definition
Femoral anatomical transepicondylar axis length	faTEA	Measured from the mediolateral line connecting the outermost edge of the epicondyle in the axial plane
Femoral surgical transepicondylar axis length	fsTEA	Measured from the mediolateral line connecting the most protruding point of the epicondyle and the medial sulcus in the axial plane
Femoral mediolateral distance	fML	Distance from the medialmost to the lateral-most aspects of the distal femur at the level of the intercondylar notch
Femoral intercondylar notch width	fNW	Mediolateral length at the widest part of the section, where the entire intercondylar fossa will be visualized in the axial plane
Femoral intercondylar notch length	fNL	Craniocaudal length at the deepest part of the section, where the entire intercondylar fossa will be visualized in the coronal plane
Femoral medial condylar length	fMCL	Craniocaudal length at the highest section where the entire intercondylar fossa is visualized in the coronal plane
Femoral lateral condylar length	fLCL	Craniocaudal length at the highest section where the entire intercondylar fossa is visualized in the coronal plane
Femoral epicondylar axis angle	fEAA	The angle between the anatomical epicondylar axis and the surgical epicondylar axis in the axial plane
Femoral posterior condylar length	fPCL/fPML	The mediolateral length passing between the two posterior condyles in the axial plane
Femoral posterior mediolateral distance	fPML	Distance between the two posteriormost points of the medial and lateral femoral condyles
Femoral anteroposterior distance	fAP	Distance from the deepest point of the trochlea to the fPML line that is tangential to the posterior femoral condyles
Femoral lateral anteroposterior distance	fLAP	Distance from the anteriormost to the posteriormost aspect of the lateral femoral condyle
Femoral medial anteroposterior distance	fMAP	Distance from the anteriormost to the posteriormost aspects of the medial femoral condyle
Femoral antero-medio-lateral distance	fAML	Distance between the two anteriormost points of the medial and lateral femoral condyles
Femoral aspect ratio	fAR	The quotient of fML and the average of the fMAP and fLAP

The sample size was calculated according to a study by Baykan et al. [12]. Using lateral condylar height length, the morphometric parameter value for women is μ_1_ ± s_1 _= 47.3 ± 4.6, and for men is μ_2 _± s_2 _= 50.5 ± 5.9. The formula used for sample size calculation was n = (r + 1) / r * ((Z_1 - α / 2_ + Z_1 - β_)^2^ s^2^) / d^2^.

Here, α is 1% level of significance, 1 - β is the power of the test, which is 80%, d = (μ_1_ - μ_2_) is the margin of error, s^2^ = (s_1_^2 ^+ s_2_^2^) / 2 is the pooled variance, and r = n_1_ / n_2_ = 1 is the equal sample size in both male and female groups. After the calculation, the sample size is n = 64. Adding 10% of nonresponse, the optimum sample size is N = n / (1 - 0.1) = 70. Therefore, n_1_ = n_2_ = 35 subjects were recruited in each gender group.

Knee MRI were taken using 3 T clinical MRI/magnetic resonance spectroscopy scanner system. Knee MRIs were taken using a 3T clinical MRI/magnetic resonance spectroscopy scanner system using a Model GE Signa 3T HDxt-32 channel MRI system (WB0427; GE HealthCare, Chicago, IL). Subjects meeting all inclusion and exclusion criteria were informed about the procedure, and informed and written consent was obtained. Subjects’ weight and height were recorded before the procedure. Subjects were positioned supine, feet first position, in the scanner, and the scan of the knee joint was done. Proton density fat-saturated MRI sequence axial, coronal, and sagittal, T1 coronal, T2, and Gradient Echo (GRE) Sagittal sequences were used for imaging knee joints with a thickness of 4 mm and an interval of 1 mm. Measurements were recorded by two observers at two different times using the same device to increase reliability. The values obtained were recorded in millimeters and degrees. The anthropometric measurements were comparatively evaluated between both sexes. Parameters mentioned in Table 1 were measured according to methods described in studies [12-15], as depicted in Figures 1-4.

Statistical analysis

The results were analyzed using descriptive statistics and comparisons among various groups. Quantitative data were summarized as mean ± standard deviation (SD). The most widely used measure of central tendency was taken as the arithmetic mean. The SD was estimated to quantify the amount of variation or dispersion of a set of data values. The unpaired t-test, also known as the independent samples t-test, was used to compare the means of two separate and independent groups in a data set to determine if there is a significant difference between them. The significance level was taken as p < 0.05.

## Results

The study included 35 subjects of both sexes, with a median age of 30 years, ranging from 19 to 75 years. The sex distribution was evenly split, with 35 men (50% of the total) and 35 women (50% of the total). Table 2 illustrates the genderwise comparison of bony parameters of the distal end of the femur.

**Table 2:** Comparison of bony parameters of distal end of femur according to gender faTEA: femoral anatomical transepicondylar axis length; fML: femoral mediolateral distance; fsTEA: femoral surgical transepicondylar axis length; fNW: femoral intercondylar notch width; fNL: femoral intercondylar notch length; fMCH: femoral medial condylar height; fLCH: femoral lateral condylar height; fEAA: femoral epicondylar axis angle; fPCL: femoral posterior condylar length; fAP: femoral anteroposterior distance; fLAP: femoral lateral anteroposterior distance; fMAP: femoral medial anteroposterior distance; fAML: femoral antero-medio-lateral distance; fAR: femoral aspect ratio

Bony parameters of distal end of femur	Gender	n	Mean	SD	95% CI	t value	p value
faTEA/fML	Male	35	81.00	3.86	79.68-82.33	12.00	<0.001
	Female	35	70.69	3.31	69.55-71.83		
fsTEA	Male	35	80.03	3.78	78.73-81.33	12.65	<0.001
	Female	35	69.42	3.22	68.31-70.52		
fNW	Male	35	23.37	1.94	22.71-24.04	4.74	<0.001
	Female	35	21.00	2.25	20.23-21.77		
fNL	Male	35	20.89	2.67	19.97-21.81	2.25	0.028
	Female	35	19.45	2.68	18.53-20.37		
fMCH	Male	35	36.21	4.35	34.71-37.70	1.08	0.283
	Female	35	34.80	6.35	32.62-36.98		
fLCH	Male	35	30.23	2.66	29.32-31.15	0.73	0.467
	Female	35	29.53	5.03	27.80-31.26		
fEAA (degree)	Male	35	5.38	0.55	5.20-5.57	3.61	0.001
	Female	35	4.72	0.94	4.39-5.04		
fPCL	Male	35	58.08	4.75	56.45-59.71	6.52	<0.001
	Female	35	51.25	3.98	49.89-52.62		
fAP	Male	35	53.28	4.62	51.69-54.87	4.57	<0.001
	Female	35	48.86	3.38	47.70-50.02		
fLAP	Male	35	57.95	2.97	56.93-58.97	4.93	<0.001
	Female	35	52.80	5.42	50.94-54.67		
fMAP	Male	35	59.44	4.90	57.76-61.13	4.89	<0.001
	Female	35	54.52	3.39	53.35-55.68		
fAML	Male	35	40.11	6.33	37.94-42.29	4.36	<0.001
	Female	35	34.38	4.54	32.83-35.94		
fAR	Male	35	1.38	0.09	1.35-1.41	3.05	0.003
	Female	35	1.32	0.08	1.30-1.35		

The bony parameters of the distal end of the femur were compared across gender groups, with detailed statistical analysis conducted on various measures (Table 3). For femoral anatomical transepicondylar axis length (faTEA)/femoral mediolateral distance (fML), femoral surgical transepicondylar axis length (fsTEA), femoral intercondylar notch width (fNW), and femoral anteroposterior (fAP), male patients exhibited statistically significant higher values compared to female patients (p < 0.001). However, for femoral intercondylar notch length (fNL), female patients showed significantly lower values than male patients (p = 0.028). No significant gender differences were observed for femoral medial condylar height (fMCH; p = 0.283) and femoral lateral condylar height (fLCH; p = 0.467). For femoral epicondylar axis angle (fEAA; degree), male patients had significantly higher values than female patients (p = 0.001). Additionally, male patients demonstrated significantly higher values than female patients for femoral posterior condylar length (fPCL), femoral lateral anteroposterior (fLAP) distance, femoral medial anteroposterior (fMAP) distance, femoral antero-medio-lateral (fAML) distance (p < 0.001).

**Table 3:** Bony parameters of distal end of femur among age group ≤30 years faTEA: femoral anatomical transepicondylar axis length; fML: femoral mediolateral distance; fsTEA: femoral surgical transepicondylar axis length; fNW: femoral intercondylar notch width; fNL: femoral intercondylar notch length; fMCH: femoral medial condylar height; fLCH: femoral lateral condylar height; fEAA: femoral epicondylar axis angle; fPCL: femoral posterior condylar length; fAP: femoral anteroposterior distance; fLAP: femoral lateral anteroposterior distance; fMAP: femoral medial anteroposterior distance; fAML: femoral antero-medio-lateral distance; fAR: femoral aspect ratio

Bony parameters of distal end of femur (age ≤30 years)	Gender	n	Mean	SD	95% CI	t value	p value
faTEA/fML	Male	11	81.41	3.61	78.99-83.83	7.26	<0.001
	Female	12	71.86	2.67	70.17-73.56		
fsTEA	Male	11	80.36	3.41	78.07-82.65	7.78	<0.001
	Female	12	70.70	2.52	69.10-72.30		
fNW	Male	11	23.29	1.72	22.14-24.44	3.39	0.003
	Female	12	20.57	2.09	19.24-21.90		
fNL	Male	11	19.83	2.99	17.82-21.84	0.66	0.516
	Female	12	20.61	2.70	18.89-22.33		
fMCH	Male	11	36.84	2.31	35.29-38.40	1.23	0.234
	Female	12	34.45	6.10	30.57-38.32		
fLCH	Male	11	29.82	1.20	29.01-30.62	0.02	0.985
	Female	12	29.85	4.66	26.89-32.80		
fEAA (degree)	Male	11	5.12	0.40	4.85-5.39	0.28	0.779
	Female	12	5.01	1.15	4.28-5.75		
fPCL	Male	11	57.98	4.00	55.29-60.67	3.59	0.002
	Female	12	51.92	4.09	49.31-54.52		
fAP	Male	11	54.09	4.88	50.81-57.37	3.05	0.006
	Female	12	48.96	3.06	47.02-50.91		
fLAP	Male	11	58.55	2.50	56.87-60.23	2.47	0.022
	Female	12	54.51	4.86	51.42-57.60		
fMAP	Male	11	60.67	2.13	59.24-62.10	3.89	0.001
	Female	12	55.98	3.43	53.80-58.16		
fAML	Male	11	39.07	2.12	37.65-40.50	2.91	0.008
	Female	12	34.83	4.39	32.03-37.62		
fAR	Male	11	1.37	0.07	1.32-1.41	1.97	0.063
	Female	12	1.31	0.08	1.25-1.36		

Table 3 shows the analysis of bony parameters of the distal end of the femur for individuals aged 30 years or younger, considering gender differences. Significant variations were observed between male and female patients in several parameters, including faTEA/fML, fsTEA, fNW, fPCL, fAP, fLAP, fMAP, and fAML (p < 0.05). In these parameters, male patients displayed higher mean values compared to female patients. However, no statistically significant gender differences were found for fNL, fMCH, fLCH, fEAA (degree), and femoral aspect ratio (fAR; p > 0.05). Notably, for parameters such as fEAA (degree) and fAR, although the differences were not statistically significant (p > 0.05), male patients tended to have slightly higher values than female patients, as shown in Table 3.

The analysis of bony parameters of the distal end of the femur among individuals aged over 30 years, categorized by gender, revealed that male patients demonstrated higher mean values compared to female patients in parameters such as faTEA/fML, fsTEA, fNW, fNL, fEAA (degree), fPCL, fAP, fLAP, fMAP, and fAML (p < 0.05). There were no statistically significant gender differences in parameters like fMCH and fLCH (p > 0.05). Male patients displayed notably higher mean values for fEAA (degree) and fAR compared to female patients, with statistical significance observed (p < 0.05), as given in Table 4.

**Table 4:** Bony parameters of the distal end of femur among age group >30 years faTEA: femoral anatomical transepicondylar axis length; fML: femoral mediolateral distance; fsTEA: femoral surgical transepicondylar axis length; fNW: femoral intercondylar notch width; fNL: femoral intercondylar notch length; fMCH: femoral medial condylar height; fLCH: femoral lateral condylar height; fEAA: femoral epicondylar axis angle; fPCL: femoral posterior condylar length; fAP: femoral anteroposterior distance; fLAP: femoral lateral anteroposterior distance; fMAP: femoral medial anteroposterior distance; fAML: femoral antero-medio-lateral distance; fAR: femoral aspect ratio

Bony parameters of the distal end of femur (age >30 years)	Gender	n	Mean	SD	95% CI	t value	p value
faTEA/fML	Male	24	80.82	4.03	79.12-82.52	9.74	<0.001
	Female	23	70.08	3.50	68.56-71.59		
fsTEA	Male	24	79.87	3.99	78.19-81.56	10.27	<0.001
	Female	23	68.75	3.39	67.28-70.21		
fNW	Male	24	23.41	2.06	22.54-24.28	3.41	0.001
	Female	23	21.22	2.34	20.21-22.23		
fNL	Male	24	21.38	2.43	20.35-22.40	3.51	0.001
	Female	23	18.85	2.51	17.76-19.93		
fMCH	Male	24	35.92	5.03	33.79-38.04	0.55	0.588
	Female	23	34.98	6.60	32.13-37.84		
fLCH	Male	24	30.43	3.12	29.11-31.74	0.84	0.406
	Female	23	29.37	5.31	27.07-31.66		
fEAA (degree)	Male	24	5.50	0.57	5.26-5.74	4.68	<0.001
	Female	23	4.56	0.80	4.22-4.91		
fPCL	Male	24	58.13	5.14	55.95-60.30	5.37	<0.001
	Female	23	50.91	3.96	49.20-52.62		
fAP	Male	24	52.91	4.55	50.99-54.83	3.42	0.001
	Female	23	48.81	3.59	47.26-50.36		
fLAP	Male	24	57.68	3.17	56.34-59.02	4.38	<0.001
	Female	23	51.91	5.58	49.50-54.33		
fMAP	Male	24	58.88	5.69	56.48-61.28	3.79	<0.001
	Female	23	53.76	3.18	52.38-55.13		
fAML	Male	24	40.59	7.51	37.42-43.76	3.51	0.001
	Female	23	34.16	4.69	32.13-36.18		
fAR	Male	24	1.39	0.10	1.35-1.43	2.33	0.024
	Female	23	1.33	0.08	1.30-1.36		

## Discussion

The knee joint's morphology differs among various ethnic groups, influenced by factors such as morphotype and sex. Ensuring optimal compatibility between the resected knee surface and the prosthesis is crucial for the long-term success of TKA. Proper placement and sizing of prosthetic components are essential for TKA longevity. Anthropometric characteristics are shaped by genetic factors, environment, sociocultural status, lifestyle, health, and functional status. Hardly any Indian studies have explored these parameters in a comprehensive manner, with most studies done on only dry bones. The current study found significant differences in distal femoral morphology between genders, which were in agreement with Murshed et al. [4], Cheng et al. [7], Guy et al. [8], Shah et al. [11], Baykan et al. [12], Hafez et al. [13], Han et al. [14], and Flores et al. [15]. The distal femur measurements of the Indian population were compared with those of Chinese, Caucasian, Arabians, Japanese, Korean, and American populations, as shown in Table 5.

**Table 5:** The distal femur measurements of Indian population compared with Chinese, Arabians, Japanese, Caucasian, Korean, and American populations All values are measured in millimeters fMAP: femoral medial anteroposterior distance; fLAP: femoral lateral anteroposterior distance; fAP: femoral anteroposterior distance; fAR: femoral aspect ratio; fML: femoral mediolateral distance; fAML: femoral antero-medio-lateral distance

Study and ethnicity	Sample and age	fMAP	fAP	fLAP	fML	fAML	fPML	fAR
Mensch and Amstutz [2] (Americans)	30 cadavers	Not reported	Not reported	Not reported	M: 82.1 ± 4.7; F: 69.9 ± 2.6; C: 76.8 ± 7.2	Not reported	Not reported	Not reported
Urabe et al. [3] (Japanese)	44 adults	Not reported	Not reported	Not reported	C: 70.6 ± 4.5	Not reported	Not reported	Not reported
Yue et al. [6] (Caucasian)	20 men and 16 women (adult)	Not reported	M: 67.5 ± 3.6; F: 59.7 ± 2.6	Not reported	M: 86.0 ± 5.6; F: 76.4 ± 4.0	Not reported	Not reported	M: 1.28 ± 0.07; F: 1.28 ± 0.06
Cheng et al. [7] (Chinese)	172 (94 men, 78 women)	F: 49.8 ± 3.2; M: 52.6 ± 2.4; combined: 51.3 ± 3.3	Not reported	F: 49.3 ± 4.1; M: 51.8 ± 3.7; combined: 50.7 ± 4.0	F: 66.8 ± 3.1; M: 74.4.6 ± 29; combined: 71.0 ± 3.0	Not reported	Not reported	F: 1.10 ± 3.6; M: 1.12 ± 3; combined: 1.11 ± 2.7
Lim et al. [9] (Korean)	115 (56 men, 59 women)	F: 56.8 ± 3.31; M: 62.7 ± 4.10	Not reported	F: 58.4 ± 3.10; M: 59.0 ± 4.01	F: 76.7 ± 3.71; M: 81.5 ± 5.70	Not reported	Not reported	F: fML/fMAP: 1.31; M: fML/fLAP: 1.35
Baykan et al. [14] (Arabians)	180 (22-60 years)	Not reported	Not reported	Not reported	F: 76.2; M: 86.2	Not reported	F: 67.9; M: 60.7	Not reported
Flores and San Juan [15] (Filipino)	675 (adults)	F: 54.9; M: 60.0; combined: 57.6 ± 4.5	Not reported	F: 54.4; M: 59.7; combined: 57.1 ± 4.6	F: 64.1; M: 74.3; combined: 69.3 ± 6.7	F: 34.6; M: 39.8; combined: 37.3 ± 4.3	F: 46.2; M: 52.9; combined: 49.6 ± 5.4	F: 1.17 ± 0.6; M: 1.24 ± 0.7; combined: 1.21 ± 0.07
Dwivedi et al. [16] (Indians)	188 women, 107 men (mean age, 69 years)	C: 56.07 ± 4.18	Not reported	C: 56.91 ± 4.78	C: 72.31 ± 5.40	Not reported	Not reported	C: 1.27 ± 0.06
Present study (North Indians)	70 (35 men, 35 women) 18 years and older	F: 54.52; M: 59.44	F: 48.86; M: 53.28	F: 52.80; M: 57.95	F: 70.69; M: 81.00	F: 34.38; M: 40.11	F: 51.25; M: 58.08	fML/fMAP: 1.30; fML/fLAP: 1.38

The current study measured the average fMAP distances as 59.44 mm in male patients and 54.52 mm in female patients, and lateral anteroposterior distances 57.95 mm in male patients and 52.80 mm in female patients. These measurements are consistent with those reported in Filipinos, Korean, and Chinese populations. However, they are smaller than the values reported for East Asian populations. Previous studies have indicated that Caucasian knees typically exhibit larger distal femur dimensions compared to Asian populations. In this study, the average mediolateral (ML) distance of the distal femur was 81 mm in male patients and 70.69 mm in female patients, similar to findings for Thai knees. A narrow fAML and a wider posterior medial lateral distance indicate a trapezoidal distal femur cross-section. Mensch and Amstutz [2] reported the fML measurements for Americans as 82.1 ± 4.7 mm for male patients, 69.9 ± 2.6 mm for female patients, and 76.8 ± 7.2 mm combined. Urabe et al. [3] provided the combined value of fML measurement for the Japanese population as 70.6 ± 4.5 mm. Murshed et al. [4] in their study examined distal femur dimensions, including the notch shape index, in male and female MRI examinations and laid emphasis on intercondylar notch variability and its relationship to gender. Yue et al. [6] detailed the measurements for Caucasians, noting that the fAP distance was 67.5 ± 3.6 mm for male patients and 59.7 ± 2.6 mm for female patients, and the fAR was recorded as 1.28 ± 0.07 for male patients and 1.28 ± 0.06 for female patients. Cheng et al. [7] suggested that morphological data should inform prosthesis design, favoring sex-specific designs. Guy et al. [8] highlighted significant differences in distal femoral morphology between men and women, noting considerable femoral component overhang in many female cases. They advocated for long-term studies to assess the clinical impact of this overhang, suggesting that sex-specific implants could reduce potential ML overhang. Measurements showed that distal femoral dimensions were generally smaller in women than in men. Lim et al. [9], using MRI, measured the mean ML width as 81.5 mm in men and 76.7 mm in women. In a study of an Arab population, Hafez et al. [13] found that the distal femoral ML width was 72.04 ± 6.6 mm, and the anteroposterior width was 68.1 ± 7.75 mm. They noted that Arab knee sizes were smaller than those of Caucasians but larger than those of Asians, with significant asymmetry in the femoral condyles and smaller dimensions in women compared to men. Shah et al. [11] in their study compared distal femoral parameters with conventional Western implants and showed significant mismatch, especially in fAR.

Han et al. [14] reported that morphometric parameters varied with age and sex, finding smaller distal femoral parameters in older individuals. They measured the transepicondylar axis (TEA) width as 83.2 mm in men and 73.9 mm in women, suggesting that ML width influenced soft tissue and bone coverage. Baykan et al. [12] in their MRI-based study measured anatomical transepicondylar axis length (aTEA; 86.2 mm in men and 76.2 mm in women), surgical transepicondylar axis (sTEA; both 83.9 mm), posterior condylar length (PCL; 67.9 mm in men and 60.7 mm in women), and NW (22.1 mm in men and 21.2 mm in women). Flores and San Juan [15] aimed to assess the fitness of existing prostheses for Filipino knees by measuring morphometric parameters of 675 knees. The results showed that while most total knee replacement prostheses can be fitted to Filipino knees, underhang on the ML aspect is commonly observed. The study suggested the need for designing prostheses specifically suited for a particular population to avoid potential mismatches. They found female knees were generally smaller than male knees [15]. The sex-specific values in the present study showed larger knee dimensions in men compared to women, consistent with findings from Asian and Arab populations. The values were similar to those reported for individuals in the Asia-Pacific region but slightly smaller than those for the Arab population [16,17]. Orthopedic surgeons must select the most suitable implant for their patient population, emphasizing the need for prosthetic manufacturers to accurately replicate patient anatomy and personalize implants. Femoral component overhang can cause soft tissue irritation, inadequate ligament balance, patellofemoral stress changes, and restricted movement due to pain. Undersizing the ML width relative to the femoral condyle can lead to postoperative complications, including hemorrhage, cancellous bone damage, and osteolysis from polyethylene wear particles. These complications can be mitigated by using sex-specific prosthesis designs.

Rotational incompatibility remains a significant cause of TKA failures. The PCL length in the axial plane has a mean internal rotation of 3° to sTEA and 5° to aTEA. Amaranath et al. [10] highlighted that the rotational alignment of the femoral component is critical for TKA success, noting a mean internal rotation of 2.3° ± 1.8° to TEA, which was higher in women. They also reported that the femoral component was typically parallel to TEA and in 3° external rotation to PCL. The angle between aTEA and sTEA, previously unexplored, was measured, with the epicondylar axis angle being 2.76° in men and 2.42° in women, indicating the importance of sTEA length for correct prosthesis rotation. In the current study, the epicondylar axis angle was 5.38° in men and 4.72° in women. Hitt et al. [18] found similar post-TKA outcomes between sexes; others propose that sex-specific prosthetic designs might be more suitable. For instance, significant differences in distal femoral morphology between men and women have been noted, with potential implications for prosthetic design and implantation.

In their study of 280 adult dried femora, Dwivedi et al. [16] documented that the mean medial condyle transverse diameter, lateral condyle transverse diameter, and intercondylar notch width were 23.65 ± 2.53, 25.25 ± 2.59, and 21.03 ± 2.53 mm, respectively. The mean NWI was 0.29 ± 0.03. The results of the present study show similarity with those of Dwivedi et al. [16]. The present study found the mean bicondylar width/fML to be 81 ± 3.86 mm in male patients and 70.69 ± 3.31 mm in female patients, with medial condylar anteroposterior diameter/fMAP and lateral condylar anteroposterior diameter/fLAP averaging 59.44 ± 4.90 mm in male patients and 54.52 ± 3.39 mm in female patients and 57.95 ± 2.97 mm in male patients and 52.80 ± 5.42 mm in female patients, respectively. ICNW/NW was 23.37 ± 1.94 mm in male patients and 21 ± 2.25 mm in female patients. Overall, the present study observed gender-based variations, with male patients generally exhibiting higher values for several parameters, such as faTEA/fML, fsTEA, fNW, fAP, fPCL, and others, indicating notable anatomical differences between the sexes. These differences were consistent across both the left and right sides of the knee joint. Statistical analysis showed that these gender-based variations were significant (p < 0.001 for most parameters), with male patients demonstrating higher mean values. However, no significant differences were noted for parameters like fNL, fMCH, fLCH, fEAA (degree), and tAR. Additionally, age-based comparisons revealed that while younger female patients had subtle variations in fAP and pH between the left and right knees, older female patients showed no significant differences in most parameters. For male patients, only fEAA (degree) showed a borderline significant change with age. Overall, the findings underscore the importance of considering gender and age in knee morphometry, which could enhance the design and effectiveness of knee prostheses. Future research with larger sample sizes and more diverse populations is essential to validate these findings further and refine prosthetic designs for better patient outcomes.

Limitations of the study

The small sample size restricts the precision of the results. A larger sample would have likely produced more accurate findings, enhancing the potential for developing improved TKA prostheses for the studied population.

## Conclusions

The present study noted significant gender differences in knee joint bony parameters. Male patients generally exhibited higher values for several parameters, such as faTEA/fML, fsTEA, fNW, fAP, and fPCL, indicating notable anatomical differences between sexes. The present research showed smaller dimensions compared to Caucasians, but values comparable to Filipinos, Koreans, and Chinese. Age-based comparisons revealed that while younger female patients exhibited subtle variations in fAP, older female patients showed no significant differences in most parameters. For male patients, only fEAA showed a borderline significant change with age. These findings highlight the importance of considering gender and age in distal femur morphometry to improve the design and effectiveness of knee prostheses.
